# Supplementary material for: Gut Microbiota, Human Blood Metabolites, and Esophageal Cancer: A Mendelian Randomization Study
Source: Genes (Basel). 2024 Jun 2;15(6):729. doi: 10.3390/genes15060729 (PMC11203100; doi:10.3390/genes15060729)
Supplement: Supplementary file 1 [file genes-15-00729-s001.zip › Additional figures.pdf]

Additional Figures

Figure S1. MR leave-one-out sensitivity analysis for Gut microbiota on esophageal cancer.

Figure S2. Scatter plots for the effect of Gut microbiota on esophageal cancer.

Figure S3. Funnel plots for the effect of Gut microbiota on esophageal cancer.

Figure S4. Forest plots for the effect of Gut microbiota on esophageal cancer.

Figure S1. MR leave-one-out sensitivity analysis for Gut microbiota on esophageal cancer.

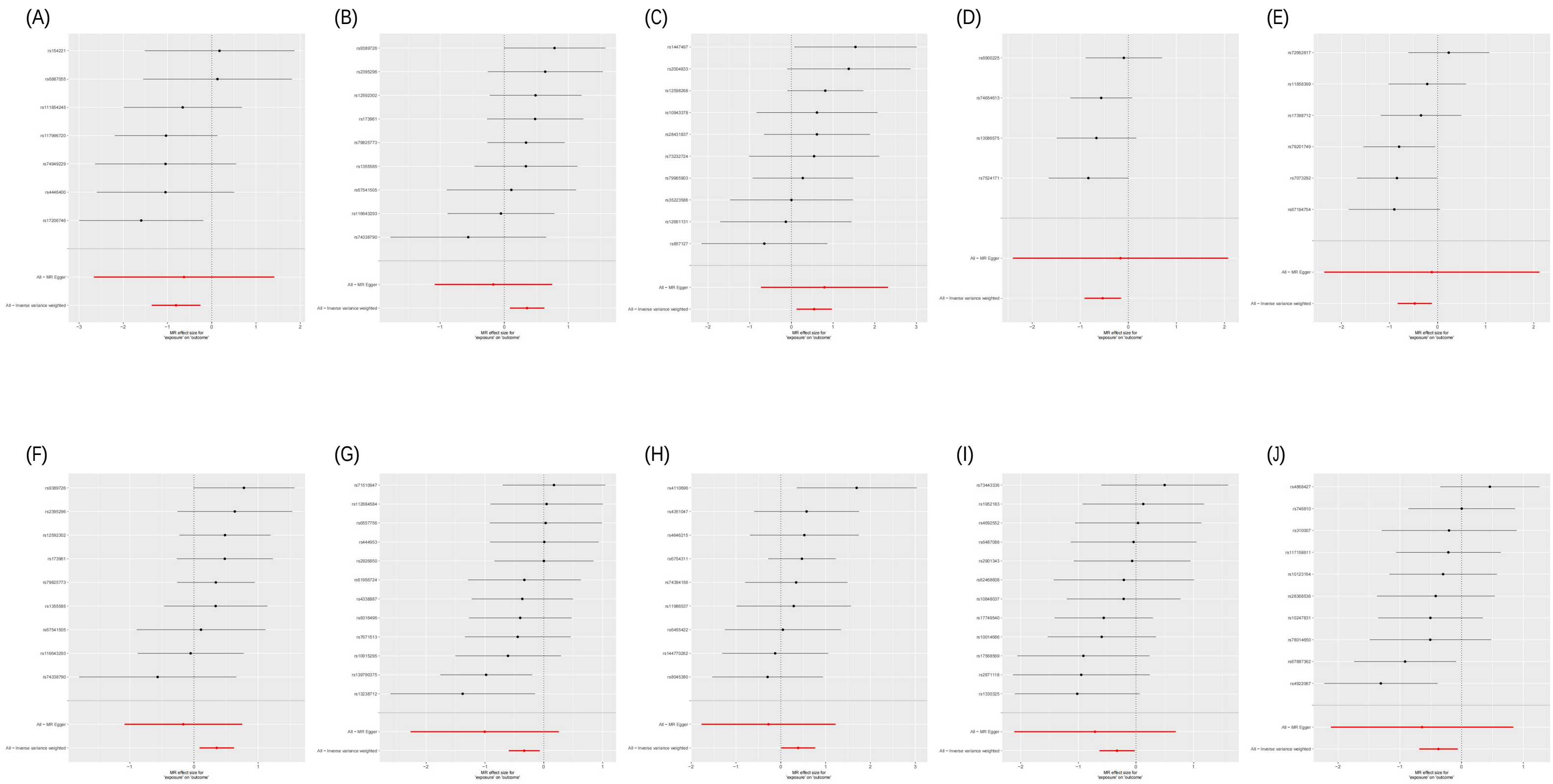

(A) Analysis for "famliy *Ruminococcaceae*" on "esophageal cancer"

(B) Analysis for "genus *Phascolarctobacterium*" on "esophageal cancer"

(C) Analysis for "phylum *Proteobacteria*" on "esophageal cancer"

(D) Analysis for "species *Streptococcus thermophilus*" on "esophageal cancer"

(E) Analysis for "species *Clostridium leptum*" on "esophageal cancer"

(F) Analysis for "species *Phascolarctobacterium succinatutens*" on "esophageal cancer"

(G) Analysis for "genus *Erysipelotrichachaceae no name*" on "esophageal cancer"

(H) Analysis for "species *Bifidobacterium adolescentis*" on "esophageal cancer"

(I) Analysis for "species *Eubacterium hallii*" on "esophageal cancer"

(J) Analysis for "species *Holdemania unclassified*" on "esophageal cancer"

Figure S2. Scatter plots for the effect of Gut microbiota on esophageal cancer.

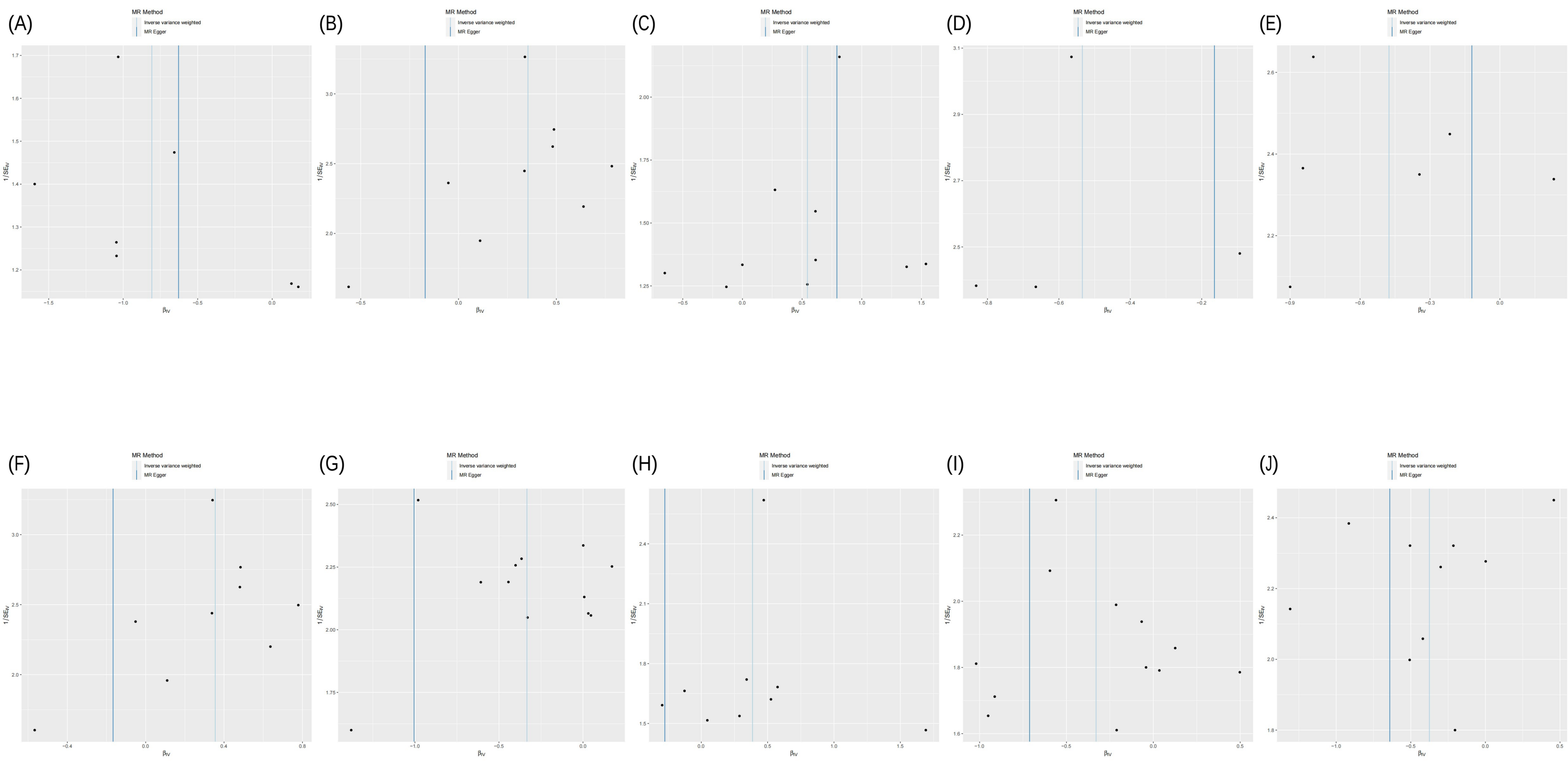

(A) Analysis for "famliy *Ruminococcaceae"* on "esophageal cancer"

(B) Analysis for "genus *Phascolarctobacterium"* on "esophageal cancer"

(C) Analysis for "phylum *Proteobacteria"* on "esophageal cancer"

(D) Analysis for "species *Streptococcus thermophilus"* on "esophageal cancer"

(E) Analysis for "species *Clostridium leptum"* on "esophageal cancer"

(F) Analysis for "species *Phascolarctobacterium succinatutens"* on "esophageal cancer"

(G) Analysis for "genus *Erysipelotrichaceae no name"* on "esophageal cancer"

(H) Analysis for "species *Bifidobacterium adolescentis"* on "esophageal cancer"

(I) Analysis for "species *Eubacterium hallii"* on "esophageal cancer"

(J) Analysis for "species *Holdemania unclassified"* on "esophageal cancer"

Figure S3. Funnel plots for the effect of Gut microbiota on esophageal cancer.

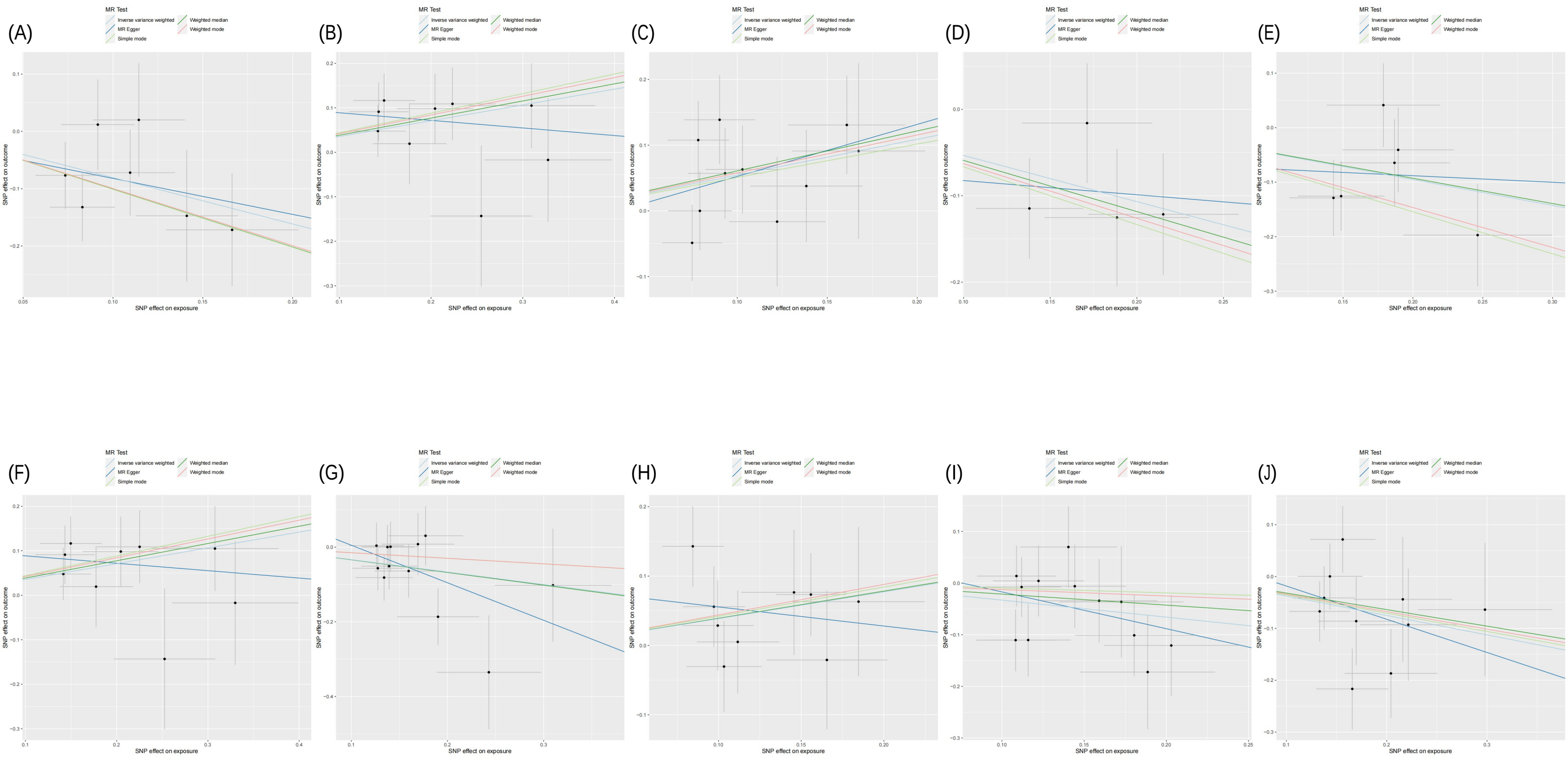

(A) Analysis for "famliy *Ruminococcaceae"* on "esophageal cancer"
(B) Analysis for "genus *Phascolarctobacterium"* on "esophageal cancer"
(C) Analysis for "phylum *Proteobacteria"* on "esophageal cancer"
(D) Analysis for "species *Streptococcus thermophilus"* on "esophageal cancer"
(E) Analysis for "species *Clostridium leptum"* on "esophageal cancer"
(F) Analysis for "species *Phascolarctobacterium succinatutens"* on "esophageal cancer"
(G) Analysis for "genus *Erysipelotrichaceae no name"* on "esophageal cancer"
(H) Analysis for "species *Bifidobacterium adolescentis"* on "esophageal cancer"
(I) Analysis for "species *Eubacterium hallii"* on "esophageal cancer"
(J) Analysis for "species *Holdemania unclassified"* on "esophageal cancer"

Figure S4. Forest plots for the effect of Gut microbiota on esophageal cancer.

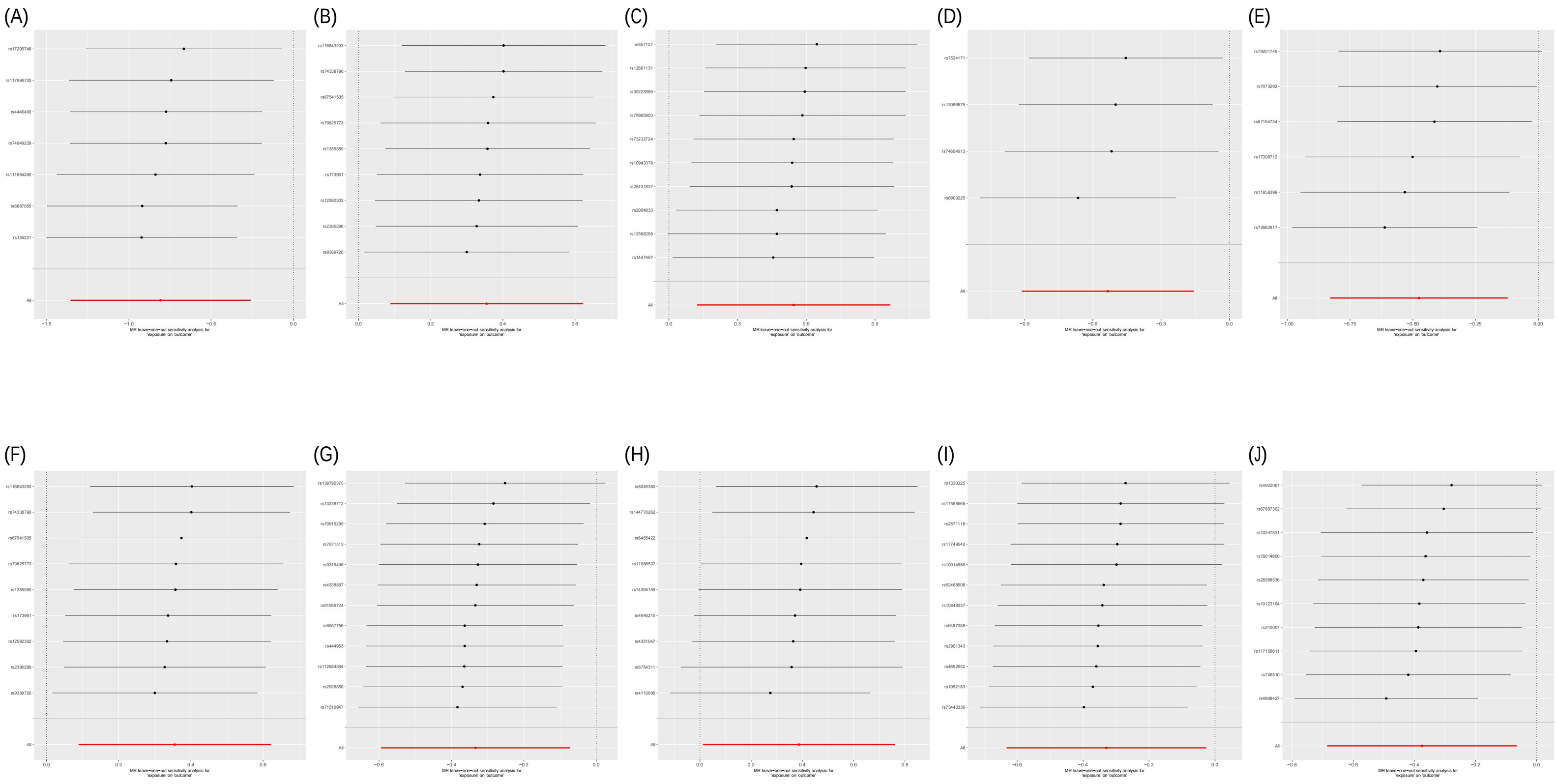

(A) Analysis for "famliy *Ruminococcaceae"* on "esophageal cancer"

(B) Analysis for "genus *Phascolarctobacterium"* on "esophageal cancer"

(C) Analysis for "phylum *Proteobacteria"* on "esophageal cancer"

(D) Analysis for "species *Streptococcus thermophilus"* on "esophageal cancer"

(E) Analysis for "species *Clostridium leptum"* on "esophageal cancer"

(F) Analysis for "species *Phascolarctobacterium succinatutens"* on "esophageal cancer"

(G) Analysis for "genus *Erysipelotrichaceae no name"* on "esophageal cancer"

(H) Analysis for "species *Bifidobacterium adolescentis"* on "esophageal cancer"

(I) Analysis for "species *Eubacterium hallii"* on "esophageal cancer"

(J) Analysis for "species *Holdemania unclassified"* on "esophageal cancer"
